# Resveratrol Inhibits the Growth of Gastric Cancer by Inducing G1 Phase Arrest and Senescence in a Sirt1-Dependent Manner

**DOI:** 10.1371/journal.pone.0070627

**Published:** 2013-11-21

**Authors:** Qing Yang, Bo Wang, Wen Zang, Xuping Wang, Zhifang Liu, Wenjuan Li, Jihui Jia

**Affiliations:** 1 Institute of Pathogen Biology, Shandong University School of Medicine, Jinan, Shandong Province, China; 2 Department of Traditional Chinese Medicine, Qilu Hospital, Shandong University, Jinan, Shandong Province, China; 3 Key Laboratory of Cardiovascular Remodeling and Function Research, Chinese Ministry of Education and Chinese Ministry of Public Health, Qilu Hospital, Shandong University, Jinan, Shandong Province, China; 4 Department of Biochemistry, Shandong University School of Medicine, Jinan, Shandong Province, China; Faculté de médecine de Nantes, France

## Abstract

Resveratrol, a naturally occurring polyphenolic compound, has been reported to exert anticancer activity by affecting diverse molecular targets. In this study, we examined the effects and the underlying mechanisms of resveratrol on gastric cancer. We found that resveratrol inhibited the proliferation of gastric cancer cells in a dose-dependent manner. At the concentration of 25 and 50 µM, resveratrol inhibited the cell viability and diminished the clonogenic potential of gastric cancer cells. Resveratrol treatment arrested gastric cancer cells in the G1 phase and led to senescence instead of apoptosis. Regulators of the cell cycle and senescence pathways, including cyclin D1, cyclin-dependent kinase (CDK4 and 6), p21 and p16, were dysregulated by resveratrol treatment. The inhibitory effects of resveratrol on gastric cancer were also verified *in vivo* using a nude mice xenograft model. Resveratrol (40 mg/kg/d) exerted inhibitory activities on gastric cancer development and significantly decreased the fractions of Ki67-positive cells in the tumor specimens from the nude mice. After resveratrol treatment, the induction of senescence and the changes in the expression of the regulators involved in the cell cycle and senescence pathways were similar to what we observed *in vitro*. However, the depletion of Sirtuin (Sirt)1 reversed the above-described effects of resveratrol both *in vitro* and *in vivo*. Our data suggest that resveratrol inhibits gastric cancer in a Sirt1-dependent manner and provide detailed evidence for the possibility of applying resveratrol in gastric cancer prevention and therapy.

## Introduction

Gastric cancer (GC) is the fourth most common cancer and the second leading cause of cancer-related mortality in the world. According to a global estimation, a total of 989,600 new GC cases were diagnosed and a minimum of 738,000 patients died from this disease in 2008, accounting for 10% of total deaths from cancer [1]. Although the incidence of GC has been declining globally, it remains high in developing countries, especially in China [1], [2]. Previous studies have revealed the intimate relationship between chronic gastritis caused by *Helicobacter pylori* infection and the development of GC [3]. Moreover, the host genetic, environmental, dietary and other factors have been implicated in the gastric oncogenic process [1]. As it is still difficult to make an early diagnosis for GC, most of the patients are diagnosed at advanced stages. Despite the improvement of conventional therapies for advanced GC, including surgery, chemotherapy and radiotherapy, the length or quality of life of patients with advanced GC is still poor [2], [4]. Therefore, the exploration of new preventive drugs or therapeutic targets of GC is urgently needed.

Consumption of fresh fruits and vegetables contributes to a decreased incidence of cancer, including GC [2], [5]. Clinical applications also suggest that some bioactive dietary molecules have the ability to inhibit multiple oncogenic steps [5]–[7]. Resveratrol (Res, 3,5,4′-trihydroxystilbene) is a naturally occurring polyphenolic compound present in almost 70 plant species, including the skin of red grapes, peanuts, berries and others [6]–[8]. Res was first reported to exert anti-tumor activities in 1997 [8]. Later reports have shown that Res imparts inhibitory effects on several types of cancers, such as colon cancer, breast cancer and lymphoma, and affects diverse molecular targets [9]–[11]. Sirtuin 1 (Sirt1), a class III nicotinamide adenine nucleotide (NAD^+^)-dependent histone/protein deacetylase, has been reported to be a key target of Res in several tumor models [9], [10]. However, some data show contrary results suggesting that Res exerts chemoprotective effects independent of Sirt1 [11]. The inhibitory effects of Res on GC and the underlying mechanism are not well studied.

In the present study, we showed that Res inhibited the proliferation of GC cells *in vitro*. Res treatment induced cell cycle arrest at the G1 phase and led to cellular senescence. These effects of Res were reverted by depletion of Sirt1. The inhibitory Sirt1-dependent activities of Res on GC cells were also verified *in vivo*. Taken together, our results indicate that Res exerts Sirt1-dependent inhibitory effects on GC and suggests a therapeutic role for Res in GC.

## Materials and Methods

### Ethics Statement

All animal studies were approved by the Ethics Committee of Shandong University School of Medicine (No. 001 in 2011 for Animal Ethics Approval) and all efforts were made to minimize suffering.

### Cell Lines, Culture Conditions and Res Treatment

Human GC cell lines AGS (obtained from Cell Resource Center, Shanghai Institute of Biochemistry and Cell Biology at the Chinese Academy of Sciences), BGC-823 and SGC-7901 (purchased from China Center for Type Culture Collection, Wuhan, China) were used in this study. The cells were cultured in F12 (AGS) or RPMI 1640 (BGC-823 and SGC-7901) containing 10% FCS, 100 units/ml penicillin and 2 mmol/L L-glutamine at 37°C in a humidified atmosphere containing 5% CO_2_. High purity Res was purchased from Sigma (St. Louis, MO, USA), dissolved in DMSO and added into the culture medium at the indicated concentration. All experiments were carried out 24 h after Res supplementation, unless otherwise indicated.

### Small Interference RNA Transfection

Chemically modified small interfering RNA (siRNA) targeting Sirt1 and control siRNA were purchased from GenePharma (Shanghai, China). The sequence of the Sirt1 siRNA was 5′-CCAUCUCUCUGUCACAAAUTT-3′. Cells were incubated overnight and then transfected with siRNA using Lipofectamine 2000 (Invitrogen, Carlsbad, CA, USA) according to the manufacturer’s protocol. Forty-eight hours after siRNA transfection, the cells were treated with 50 µM Res for 24 h before further study.

### Cell Viability Assay

Proliferation was assessed using the Cell Titer 96 ® AQueous One Solution Cell Proliferation Assay (Promega, Madison, WI, USA). Briefly, 2×10^3^ cells were seeded in a 96-well plate and allowed to grow for 24 h. Twenty-four hours later, 20 µl MTS (3-(4,5-dimethylthiazol-2-yl)-5-(3-carboxy-methoxyphenyl)-2-(4-sulfophenyl)-2H-tetrazolium) was added to each well. After incubation for 3 h at 37°C, the absorbance at 490 nm was recorded on a Varioskan Flash Multiplate Reader (Thermo Scientific, Waltham, MA, USA). Cell viability was calculated by the following formula: relative cell viability = (average absorbance of treated group - average absorbance of blank)/(average absorbance of control group- average absorbance of blank). Assays were performed in triplicate and repeated three times.

### Colony Formation Assay

Cells were seeded into 6-well plates (300 or 500 cells per well) and incubated for 10 days until the colonies were large enough to be clearly discerned. The cells were fixed with methanol and stained with crystal violet, and the number of colonies with more than 50 cells was counted manually. Experiments was performed in triplicate and repeated three times.

### Cell Cycle Analysis

Cells were harvested, fixed with pre-cooled 70% ethanol at 4°C overnight and then stained with propidium iodide (Beyotime, Jiangsu, China) containing RNase A at 37°C for 30 min in the dark. The cell cycle distribution was determined using a flow cytometer (BD Biosciences, San Jose, CA, USA) and the data were analyzed with Multicycle software (Phoenix Flow Systems, San Diego, CA, USA). Experiments were performed independently three times.

### Apoptosis Assay

Detection and quantitation of apoptosis were performed by labeling of DNA strand breaks using an In Situ Cell Death Detection Kit, TMR red (Roche Applied Science, Basel, Switzerland). Cells or paraffin sections of xenografts were labeled with TUNEL according to the manufacturer’s instructions. For cells, treatment with 0.2 mM H_2_O_2_ for 12 h served as the positive control. For paraffin sections, positive controls were purchased from Millipore (Billerica, MA, USA). The nuclei were counterstained with DAPI (Beyotime) and the slides or sections were imaged by a fluorescence microscopy (Olympus, Tokyo, Japan) using cellSens Dimension software. Experiments were performed independently three times.

### β-Galactosidase Staining

Senescence was assessed using a Senescence β-Galactosidase Staining Kit (Beyotime). Briefly, cells or frozen sections of xenografts were fixed and then incubated with freshly prepared β-Galactosidase (β-Gal) staining solution at 37°C overnight. Experiments were performed independently three times.

### Stable Lentiviral-short Hairpin RNA (shRNA) GC Cells

Lentiviral vectors containing control or Sirt1 shRNA were constructed by GenePharma and used to transfect BGC-823 cells. Efficient knockdown of Sirt1 was verified by western blot. For stable transduction, control-lentivirus-infected or Sirt1 shRNA-lentivirus-infected BGC-823 cells were cultured in complete medium supplied with 2 µg/ml puromycin for four weeks and regarded as LV-C and LV-S, respectively.

### Nude Mice Xenograft Model

Female athymic BALB/c nude mice (6∼8 weeks) were purchased from the Peking University (Beijing, China) and were maintained under specific pathogen-free conditions at the Key Laboratory of Cardiovascular Remodeling and Function Research, Qilu hospital, Shandong University. The mice were randomly divided into four groups (8 mice for each group): group I and II, BGC-823 cells (1×10^6^ cells per injection in 0.1 ml PBS) were subcutaneously injected into the flank region of the nude mice; group III, LV-C (BGC-823 cells) was injected; and group IV, LV-S (BGC-823 cells) was injected. After implantation, the nude mice from groups II, III and IV were treated with Res (40 mg kg^−1^ in 0.1 ml vegetable oil, administrated by gavage, once daily). The subcutaneous tumor size was measured with a caliper, and the tumor volumes were calculated by the formula (length) × (width^2^)/2. The mice were sacrificed four weeks later. The tumors were harvested and processed for western blot and immunochemical studies.

### Real Time-quantitative PCR (RT-QPCR)

Total RNA in cells was isolated using TRIzol reagent (Invitrogen) and converted into cDNA using the PrimeScript™ RT reagent kit (Takara, Tokyo, Japan). RT-QPCR was performed for genes, including cyclin D1, cyclin-dependent kinase 4 (CDK4), p21 and β-actin as previously described [12]. The sequences of the amplification primers are listed in Table S1. The mRNA expression of cyclin D1, CDK4 and p21 was normalized to β-actin relative to the control using the 2^−ΔΔCt^ method. Each experiment was repeated in triplicate.

### Western Blot

Total protein from the cells or the tumor specimens was extracted with RIPA Lysing Buffer (Beyotime) as described [12]. The protein concentration was determined using the BCA Protein Assay Kit (Pierce, Rockford, IL, USA). The membrane was probed with antibodies against Sirt1 (Abcam, Cambridge, MA, USA), bcl-2, bax, caspase-3, cyclin D1, CDK4 and 6 (Cell Signaling, Danvers, MA, USA), p16 and p21 (Santa Cruz Biotechnology, Santa Cruz, CA, USA). The secondary antibody used was a horseradish peroxidase (HRP)-conjugated anti-rabbit or mouse antibody. The protein bands were visualized using an ECL system (Pierce). β-actin (Cell Signaling) served as a loading control.

### Immunohistochemistry

The tumor specimens from the nude mice were deparaffinized, rehydrated and antigen retrieved. The sections were incubated with an antibody against Ki67 (1∶500, Abcam) overnight at 4°C. HRP-conjugated anti- rabbit IgG and DAB staining were employed to visualize the Ki67 antibody. The slides were finally counterstained with hematoxylin. The slides were imaged by an Olympus light microscope (Tokyo, Japan) using cellSens Dimension software. The Ki67-positive cells were counted manually and the percent of Ki67-positive cells were assessed per fields. Five separate fields were counted for each section.

### Statistical Analyses

The data were expressed as the mean ± SD. The statistical analyses were performed using Statistical Package for the Social Sciences (SPSS, version 16.0, Chicago, IL, USA) by one-way ANOVA with Tukey’s post-hoc test. P-values less than 0.05 were considered statistically significant.

## Results

### Res Inhibits the Viability of GC cells in a Sirt1-dependent Manner

**Figure 1:**
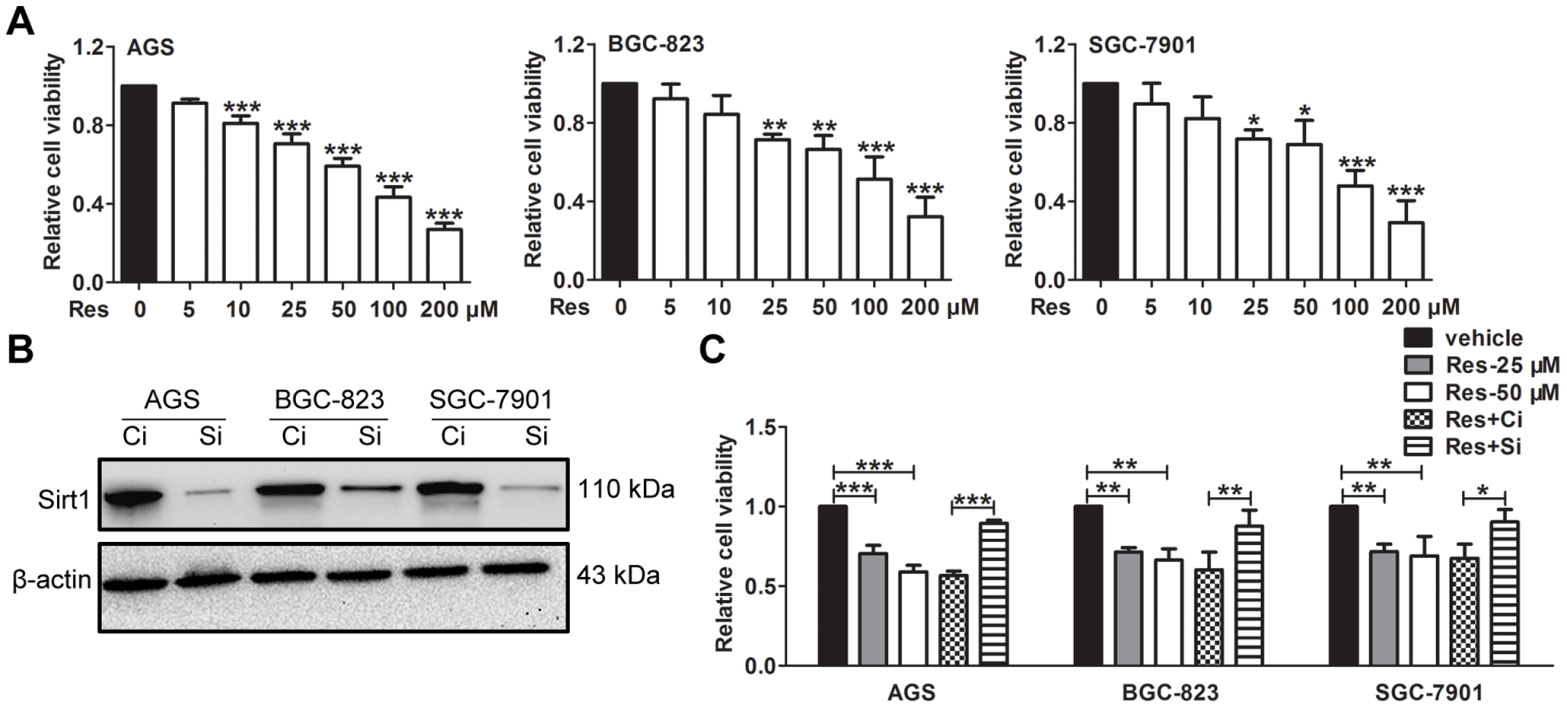
Res inhibits the viability of GC cells in a Sirt1-dependent manner. (A) GC cells were treated with or without Res at the indicated concentration for 24 h. The cell viability was then measured by MTS assays. (B) The expression of Sirt1 at 72 h after control or specific siRNA transfection was determined by western blot. ‘Ci’ represents the control siRNA, and ‘Si’ represents the Sirt1 siRNA. (C) Forty-eight hours after siRNA transfection, GC cells were treated with 50 µM Res for 24 h. Then the MTS assays were performed. The data represent the mean ± SD, * represents P<0.05, ** represents P<0.01 and *** represents P<0.001.

Three GC cell lines (AGS, BGC-823 and SGC-7901) were examined in our experiments. Culturing the cells in vehicle (0.1% DMSO) did not affect cell viability (data not shown). However, when treated with different concentrations of Res for 24 h, cell proliferation was dose-dependently inhibited at 25, 50, 100 and 200 µM Res (Figure 1A). Of note, proliferation of AGS was obviously inhibited when treated with 10 µM Res, which was not observed in the other two cell lines (Figure 1A). In all the three cell lines, the presence of 100 µM Res was sufficient to induce more than 50% growth inhibition (56.78% for AGS, 54.87% for BGC-823 and 52.16% for SGC-7901, respectively). Therefore, 25 and 50 µM Res were used for subsequent experiments. To determine the functional role of Sirt1 in Res treatment, we knocked down Sirt1 expression using a specific siRNA. The efficient inhibition of Sirt1 expression was verified by western blot (Figure 1B). The results of the MTS assay showed that in Sirt1-depleted cells, Res did not inhibit cell proliferation (Figure 1C). These data indicate that Res is able to inhibit the proliferation of GC cells and that the inhibitory effect can be rescued by Sirt1 depletion.

### Res Diminishes the Clonogenic Potential of GC cells in a Sirt1-dependent Manner

**Figure 2:**
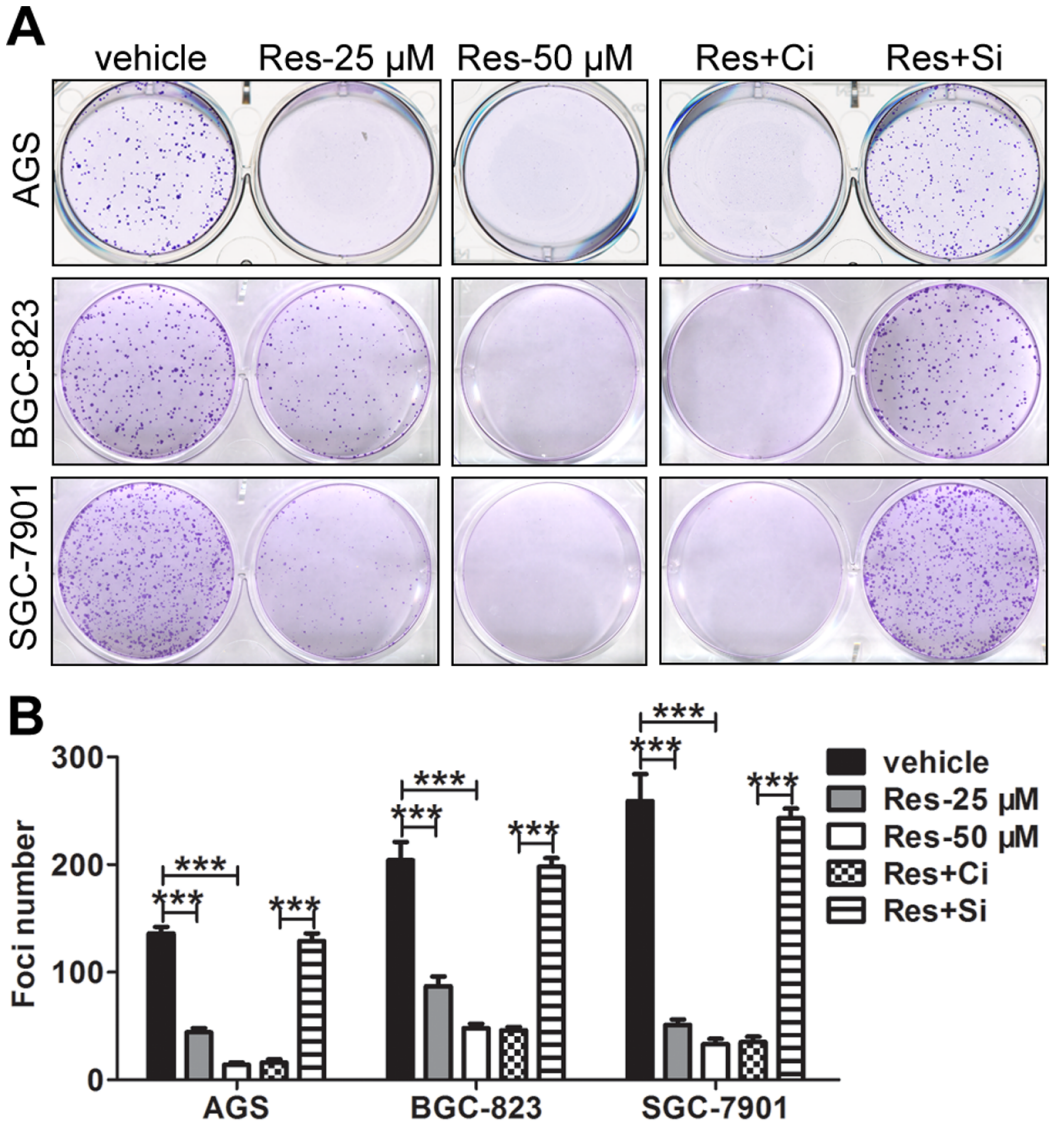
Res diminishes the clonogenic potential of GC cells in a Sirt1-dependent manner. The colony formation experiments were performed in triplicate and repeated three times. The representative graphs are shown in (A). The quantitative analysis is demonstrated as a histogram in (B). ‘Ci’ represents the control siRNA, and ‘Si’ represents the Sirt1 siRNA. The data represent the mean ± SD, the statistical results are indicated by asterisks and *** represents P<0.001.

For tumor cells, colony formation has been found to be a more sensitive parameter than viability to assess the effect of a drug. Thus, efforts were then undertaken to determine the effect of Res on the clonogenic potential of GC cells. Res, at the concentration of 25 µM, led to a significant reduction in foci numbers as well as sizes in GC cells. After treatment with 50 µM Res, the clonogenic potential diminished further. However, knockdown of Sirt1 prior to Res treatment increased the number of foci to a level comparable to the vehicle group (Figure 2).

### Res Induces the Accumulation of GC Cells in the G1 Phase in a Sirt1-dependent Manner

**Figure 3:**
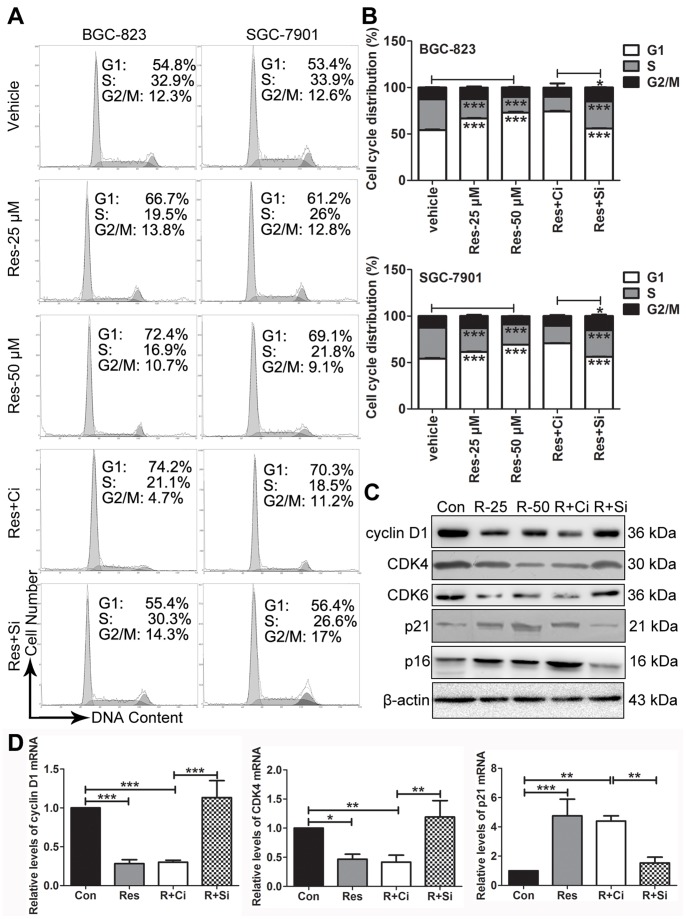
Res induces the accumulation of GC cells in G1 phase in a Sirt1-dependent manner. The cell cycle distribution of the cells was analyzed by flow cytometry. The representative graphs are shown in (A). The quantitative analysis is demonstrated as histograms in (B). (C) The protein levels of regulators of cell cycle were detected by western blot. (D) The mRNA levels of regulators of cell cycle were detected by RT-QPCR. ‘Con’ represents vehicle treatment, ‘R’ represents resveratrol, ‘Ci’ represents the control siRNA, and ‘Si’ represents the Sirt1 siRNA. The data represent the mean ± SD, * represents P<0.05 and *** represents P<0.001.

To examine the inhibitory effect of Res on GC cells, we performed cell cycle analysis. We observed that 25 and 50 µM Res increased the proportion of cells in the G1 phase in both BGC-823 and SGC-7901 cells (Figures 3A and 3B). The induction of the G1 phase arrest by Res was also dose-dependent. Res at a concentration of 50 µM showed a stronger effect than it did at 25 µM. The increase of the number of cells in the G1 phase was accompanied by a decrease in the cell populations mainly in the S phases. The depletion of Sirt1 prior to Res treatment diminished the G1 phase induction of Res (Figures 3A and 3B). To corroborate the above results, we examined the expression of cell cycle regulators of the G1 phase, including cyclin D1, CDK4, CDK6, p21 and p16 in BGC-823 cells. As shown in Figure 3C, in Res-treated BGC-823 cells, the protein levels of activators of the G1/S transition (cyclin D1, CDK4 and CDK6) decreased, while the protein levels of inhibitors of CDKs (CDKIs) (p21 and p16) increased. In contrast, these changes were not found in Sirt1-depleted BGC-823 cells when they were treated with 50 µM Res. Similar results were obtained from the RT-QPCR of cyclin D1, CDK4 and p21 (Figure 3D).

The lack of sub-G1 cells indicated that Res treatment did not trigger apoptosis in the GC cells (Figure 3A), which was consistent with results of TUNEL labeling experiments from BGC-823 cells (Figure S1A). The protein levels of apoptosis-related molecules, such as bcl-2, bax and caspase-3, from each group were comparable (Figure S1B). Taken together, our results show that Res induces G1 phase arrest in GC cells in a Sirt1-dependent manner and does not induce apoptosis.

### Res Induces Senescence of GC Cells in a Sirt1-dependent Manner

**Figure 4:**
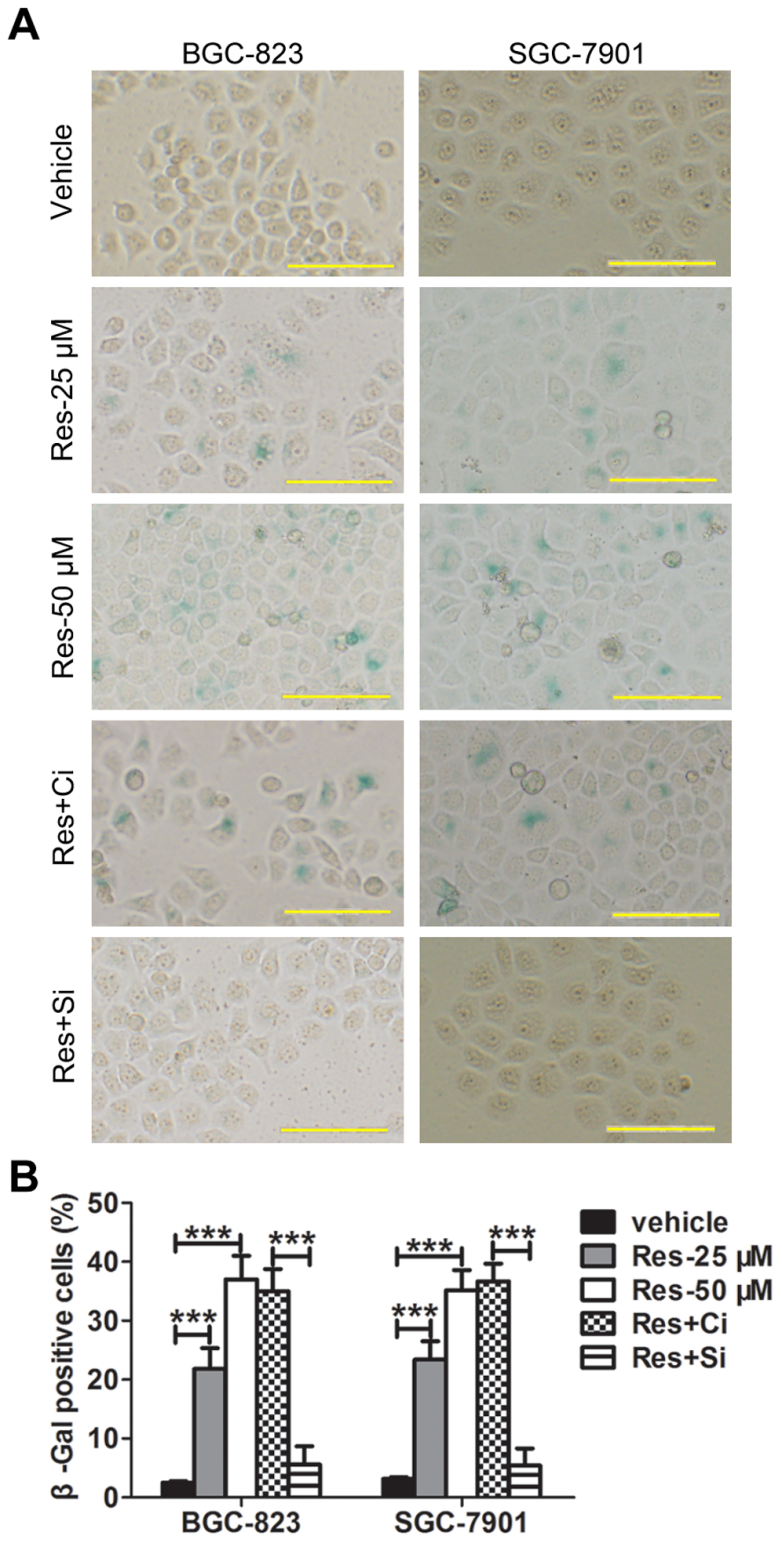
Res induces senescence of GC cells in a Sirt1-dependent manner. β-Gal staining was performed to detect senescent cells. The representative graphs are shown in (A). Magnification: × 200, bar for 100 µm. The quantitative analysis is demonstrated as a histogram in (B). ‘Ci’ represents the control siRNA and ‘Si’ represents the Sirt1 siRNA. The data represent the mean ± SD, the statistical results are indicated by asterisks and *** represents P<0.001.

In our hands, Res results in growth arrest rather than increased apoptosis. Therefore, we made further efforts to explore whether Res could induce senescence in GC cells. β-Gal staining, a specific marker for mammalian senescent cells, was used. After Res treatment, we found increased fractions of cells were stained with β-Gal. However, the pretreatment with Sirt1 siRNA blocked Res-induced cellular senescence (Figure 4). Similar results were obtained from both BGC-823 and SGC-7901 cells, which suggests that Res dependents on Sirt1 to induce cellular senescence in GC cells.

### Res Inhibits Tumor Growth in vivo in a Sirt1-dependent Manner

**Figure 5:**
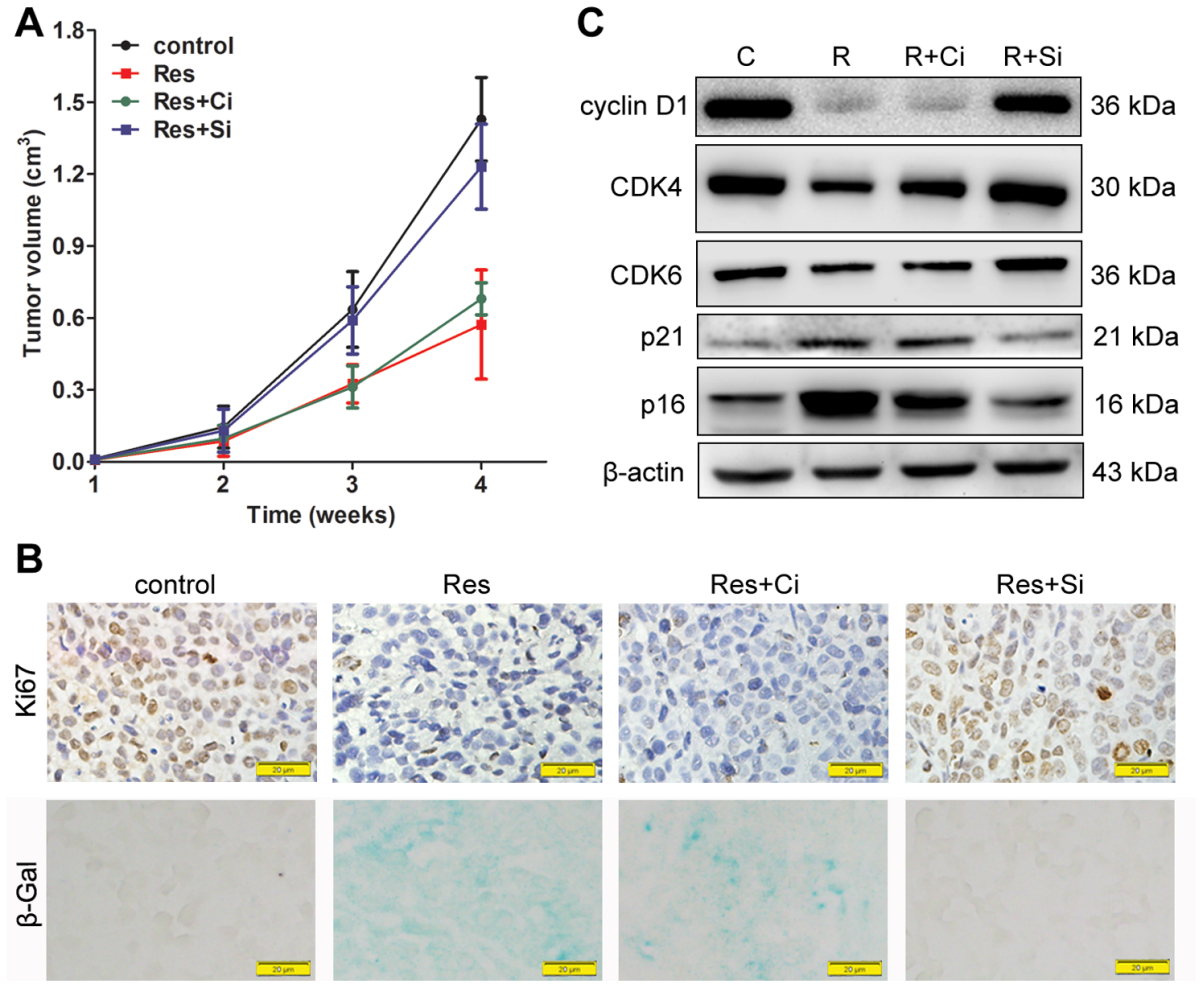
Res inhibits tumor growth *in vivo* in a Sirt1-dependent manner. (A) Nude mice were randomly divided into four groups and there were 8 mice for each group. BGC-823 cells (1×10^6^) with different treatments were injected subcutaneously into each group. After implantation, the tumor size was measured weekly. The tumor volumes were calculated by the formula (length) × (width^2^)/2. (B) The proliferating cells in the tumor specimens from the different groups were detected by Ki67 immunochemistry. Senescence was indicated by β-Gal staining. The representative graphs are shown. Magnification: × 400, bar for 20 µm. (C) Total protein from the tumor specimens was extracted, and the expression of the regulators of cell cycle were detected by western blot. ‘C’ represents control, ‘R’ represents resveratrol, ‘Ci’ represents the stable control lentiviral-shRNA BGC-823 cells, and ‘Si’ represents the stable Sirt1 lentiviral-shRNA BGC-823 cells.

Next, additional studies were performed to determine the effects of Res on BGC-823 xenografts growth in nude mice. For the *in vivo* study, stable transduced lentiviral-shRNA BGC-823 cells were used. Of the four Sirt1 shRNA-lentiviruses, LV-1 and LV-4 exerted obvious silencing effects on Sirt1 expression (Figure S2). After four weeks of screening, the expression of Sirt1 was maintained at the decreased levels in the stable lentiviral-shRNA BGC-823 cells (Figure S2). Stable LV-1 lentiviral-shRNA BGC-823 cells were used in subsequent xenografts studies because of the stronger inhibitory effects on Sirt1 expression compared to the LV-4. All of the animals survived to the end of our experiments, and no obvious difference was found in their body weight (data not shown). Four weeks after implantation, measurements of the tumor volumes indicated that Res treatment significantly reduced the growth of BGC-823 xenografts (Res vs control: 0.5728±0.2276 cm^3^ vs 1.4288±0.1741 cm^3^, P<0.001). Stable transduction of the control shRNA-lentivirus did not significantly affect the tumor volume (Res vs Res+Ci: 0.5728±0.2276 cm^3^ vs 0.68±0.0672 cm^3^, P = 0.603). However, in the Sirt1-depleted xenografts, the inhibitory effects of Res on tumor growth were rescued (Res+Si vs Res+Ci: 1.2313±0.1777 cm^3^ vs 0.68±0.0672 cm^3^, P<0.001, Res+Si vs control: 1.2313±0.1777 cm^3^ vs 1.4288±0.1741 cm^3^, P = 0.123) (Figure 5A). In the Res-treated xenografts, the proliferation marker, Ki67, decreased significantly (Figure 5B) (% of Ki67-positive cells, Res vs control: 3±1.8 vs 44.67±3.79, P<0.001). Senescence was observed in xenografts from Res-treated mice indicated by β-Gal staining (Figure 5B). However, no obvious apoptosis was induced by Res in the xenografts (Figure S1D) and no changes were observed in the regulators of apoptosis, such as bcl-2, bax and caspase-3 (Figure S1C). These results were consistent with the *in vitro* experiments. Moreover, the changes in the expression of the regulators of cell cycle, including cyclin D1, CDK4, CDK6, p21 and p16 were similar to what we observed *in vitro* (Figure 5C). All the changes observed in Ki67, β-Gal and the cell cycle regulators were reversed by Sirt1 depletion (Figures 5B and C) (% of Ki67-positive cells, Res+Si vs Res+Ci: 46.32±6.03 vs 2.65±1.53, P<0.001, Res+Si vs control: 46.32±6.03 vs 44.67±3.79, P = 0.946).

## Discussion

Res, a natural polyphenol, is currently being evaluated as a promising anticancer agent. Although it has been proven to impart antiproliferative effects against several cancer types both in cell culture and xenograft models [9]–[11], its chemoprevention effects on GC and the underlying mechanism have not been well studied. In this study, we demonstrated that Res inhibited the proliferation of GC cell lines (AGS, BGC-823 and SGC-7901). The anti-growth activity was observed after the cells were treated with 25 µM Res for 24 h, and the inhibitory effect was dose-dependent. At a concentration of 50 µM Res, the inhibition ratios for these three cell lines were 41%, 34% and 32%, respectively. When the concentration of Res increased further, the inhibitory effects were strengthened. Both 100 and 200 µM Res inhibited growth by more than 50% compared to the vehicle group. In addition, the higher dose of Res is too great to achieve *in vivo* and thus makes no sense in a clinical setting [13], [14]. Therefore, the two lower effective concentrations of 25 and 50 µM Res were used in the subsequent studies. The growth inhibition activity of Res appears to be cell-specific, as the IC50 differs according to the cell type and varies from 27 µM to 180 µM [9], [10], [15], [16]. Some of these studies have also shown that Res exerts the inhibitory effects in a time-dependent manner [15], [16]. The concentration and duration of Res treatment used in our study were consistent with the majority of reports from other groups [8], [9], [16]. For the *in vivo* study, the administration method we used is gavage because this is the method that is commonly used in mice as an alternative to intraperitoneal injection [15], [17]. In addition, studies in mice have revealed that Res is absorbed efficiently after oral administration and can be found throughout the body [17], [18]. When used *in vivo*, Res significantly decreased the proliferation of cells as characterized by Ki67 expression, which is consistent with the results from our *in vitro* experiments.

Res can slow down the proliferation of cancer cells through diverse mechanisms, among which, cell cycle regulation is an important one [9], [15], [19]. Our *in vitro* data demonstrated that the treatment of GC cells with Res induces G1 phase arrest. The G1 phase is the first of the four phases of the cell cycle that takes place in eukaryotic cell division. G1 is a particularly important cell cycle phase because it is the point at which a cell commits to a round of division. Progression through G1 phase requires the formation and action of cyclin D-CDK4 or -CDK6 complexes. These complexes then phosphorylate retinoblastoma, which leads to the release of the E2F transcription factors and the downstream gene transcription involved in S phase progression. The activities of the cyclin D-CDK complexes are regulated by upstream inhibitors, including members of the INK (p15, p16 and p18) and CIP families (p21, p27 and p57) [20], [21]. Along the same line, accompanied with the G1 phase arrest, we observed the downregulation of cyclin D1, CDK4 and CDK6 and the upregulation of p21 and p16 in Res-treated GC cells. Our *in vivo* results of the expression levels of these cell cycle regulators in the tumor specimens are consistent with the *in vitro* results. Our results are also consistent with those of previous studies [15], although some others have reported that S phase arrest is induced by Res [9], [19]. Persistence of growth arrest may lead to apoptosis or senescence in cells. Because both p21 and p16 signaling pathways participate in senescence progression mediated by various types of stress [22], [23], we performed β-Gal staining. Res treatment significantly increased the frequency of senescent GC cells in a dose-dependent manner. The cellular senescence program represents an important barrier against cancer initiation and development [24], [25]. Although previous studies show that, through the attenuation of oxidative stress and the amelioration of metabolism, Res exerts anti-aging effects both *in vitro* and *in vivo* [26]–[28], the opposite effects have been shown in cancer. Res has been found to induce senescence-like growth inhibition in several types of cancers [29], [30]. The dual role of Res in cellular senescence, retarding aging in normal tissues and accelerating senescence in tumors, makes it an ideal candidate for cancer prevention and treatment.

Apoptosis is another common effect that is usually observed in Res-treated cancer cells [9], [11], [15], [16]. Experimental evidence indicates that apoptosis can be mediated by several different pathways and numerous regulatory molecules. Among these regulators, the proteins comprising the bcl-2 family are important. There are both anti-apoptotic proteins (bcl-2, bcl-xl) and pro-apoptotic proteins (bax, bad, bak and bcl-xs) in this family. An increase of the pro-apoptotic bax/anti-apoptotic bcl-2 ratio enhances the permeability of the mitochondrial membrane, causes the release of cytochrome c from mitochondria to cytosol and, in turn, results in the activation of caspase-9 and the downstream target caspase-3 [31]. Despite this intrinsic apoptotic pathway, the pro-apoptotic ligands, such as FasL, by binding to their receptors, cause the activation of caspase-8 and downstream effectors, such as caspase-3. The activation of effector caspases induces the cleavage of many cellular substrates and directly causes apoptosis [31]–[33]. Although numerous previous reports indicated that apoptosis was responsible for Res-induced growth inhibition, we did not observe obvious apoptosis in Res-treated GC cells. This absence of apoptosis may be due to the concentration and duration of Res treatment adapted in our experiment because studies from different groups show that Res exerts dosage- and duration-dependent effects [34], [35]. In addition, as the effects of Res are also cell-specific [36]–[38], the GC cells used in our study may be resistant to Res-induced apoptosis.

Res has multiple targets; among which, the class III NAD^+^-dependent deacetylase Sirt1 is the most frequently studied. Although a biochemical study indicated that Res was not a direct activator of Sirt1 [39], Res has still been regarded as a classic agonist of Sirt1. Numerous studies show that Res exerts diverse effects in different models in a Sirt1-dependent manner [40], [41]. In our experiment, Sirt1-depletion reversed the growth inhibition of Res both *in vitro* and *in vivo*. The G1 phase arrest and senescence induced by Res treatment were rescued by Sirt1-depletion. These results indicate that the inhibition effects of Res on GC are dependent on Sirt1. According to the previous studies, Sirt1 may regulate gene expression through two different mechanisms. First, directly, Sirt1 directly mediates the deacetylation of a protein and then enhances its ubiquitination and subsequent ubiquitin proteasome degradation [42]. Second, indirectly, Sirt1 exerts deacetylase activity influencing the chromatin confirmation or repressing the activities of transcription factors and then regulates the transcription of genes [43]–[45]. As the molecules (cyclin D1, CDK4, CDK6, p21 and p16) detected in our experiments have not been reported as the direct targets of Sirt1, we speculated that Sirt1 might regulate the expression of these genes mainly through the indirectly mechanism. The results of RT-QPCR also support this hypothesis.

Taken together, our findings suggest that Res inhibits both the proliferation of GC cells *in vitro* and the growth of xenografts *in vivo*. Res treatment induces G1 phase arrest in GC cells and leads to senescence instead of apoptosis. Sirt1-depletion rescues the above-described effects of Res both *in vitro* and *in vivo*. Our work indicates that Res inhibits GC in a Sirt1-dependent manner and provides detailed evidence for the possibility of applying Res in GC prevention and therapy.

## Supporting Information

Figure S1 **Res exerts no effects on apoptosis of BGC-823 cells.** (A) Apoptosis was indicated by TUNEL labelling (red) and BGC-823 cells were counterstained with DAPI (blue). Treatment with H_2_O_2_ served as the positive control. Original magnification: × 200. Scale bars, 50 µm. (B) Regulators of apoptosis in BGC-823 cells, including bcl-2, bax and caspase-3 were analyzed by western blot. (C) Regulators of apoptosis in the xenografts, including bcl-2, bax and caspase-3 were analyzed by western blot. For detection of cleaved caspase-3, BGC-823 cells treated with H_2_O_2_ served as the positive control (shown in the right panel). (D) Apoptosis in xenografts was detected by TUNEL labeling (red) and the sections were counterstained with DAPI (blue). Sections from female rodent mammary gland obtained 3∼5 after weaning of rat pups (Millipore) served as the positive control. Original magnification: × 200. Scale bars, 50 µm. ‘R’ represents resveratrol, ‘Ci’ represents the control siRNA, and ‘Si’ represents the Sirt1 siRNA.
(TIF)

Figure S2 **Knockdown of Sirt1 by shRNA-lentivirus.** (A) BGC-823 cells were transfected with control or Sirt1-specific shRNA-lentiviruses. The transfection efficiency was evaluated with a fluorescence microscope. At a multiplicity of infection (MOI) of 50, more than 90% of the cells were transfected with shRNA-lentivirus. After four weeks of screening with puromycin, all of the living cells were transduced. Magnification: × 100, bar for 200 µm. (B) The cells were harvested 4 days after infection and 28 days after puromycin screening. The silencing efficiency of Sirt1 was verified by western blot.
(TIF)

Table S1 **Primers for the RT-QPCR experiments performed in this study.**
(DOC)
