# Supplementary figures and images for: Resveratrol Inhibits the Growth of Gastric Cancer by Inducing G1 Phase Arrest and Senescence in a Sirt1-Dependent Manner
Source: PLoS One. 2013 Nov 21;8(11):e70627. doi: 10.1371/journal.pone.0070627 (PMC3836800; doi:10.1371/journal.pone.0070627)

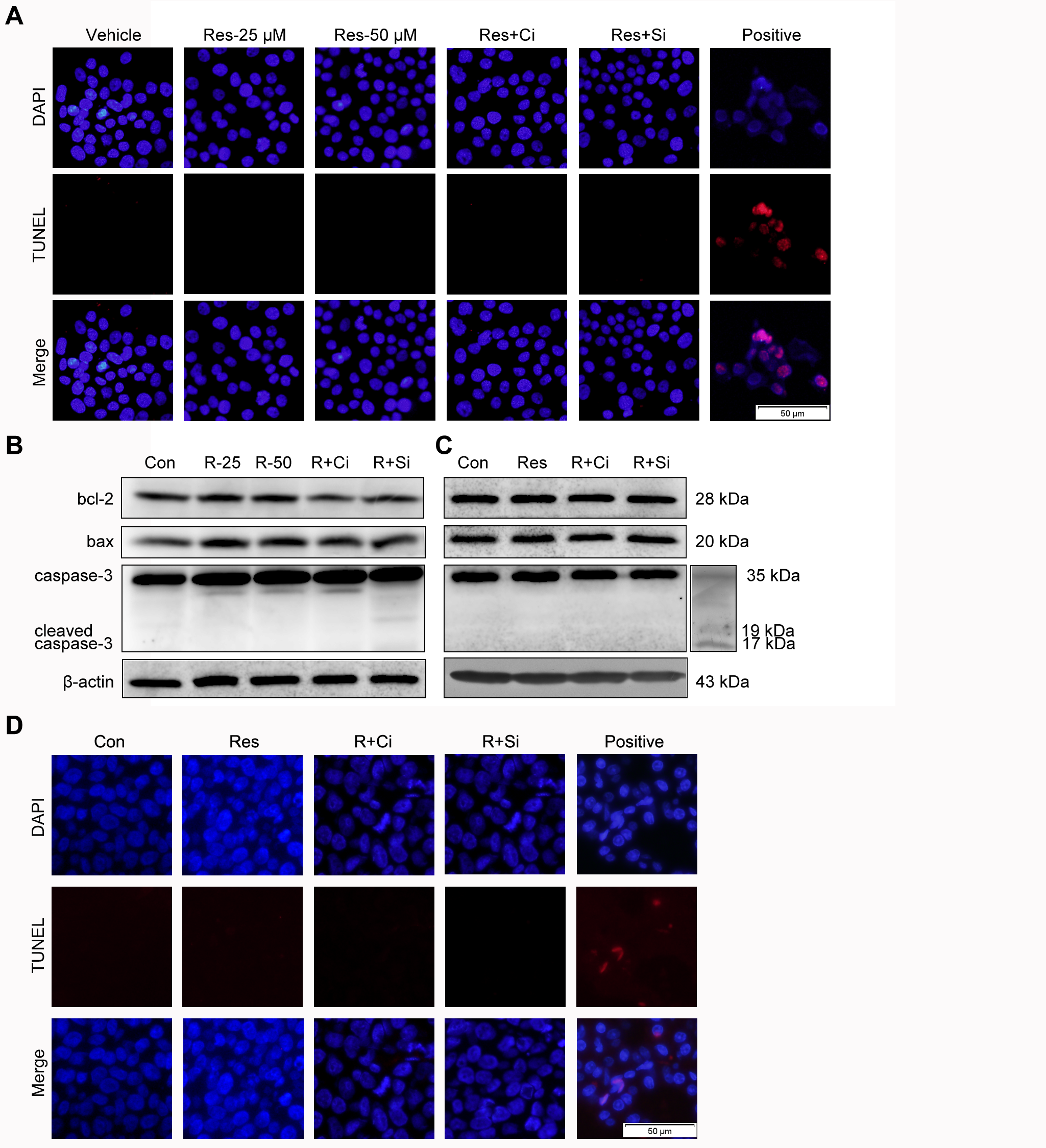

Supplement: Figure S1 — Res exerts no effects on apoptosis of BGC-823 cells. (A) Apoptosis was indicated by TUNEL labelling (red) and BGC-823 cells were counterstained with DAPI (blue). Treatment with H2O2 served as the positive control. Original magnification: × 200. Scale bars, 50 µm. (B) Regulators of apoptosis in BGC-823 cells, including bcl-2, bax and caspase-3 were analyzed by western blot. (C) Regulators of apoptosis in the xenografts, including bcl-2, bax and caspase-3 were analyzed by western blot. For detection of cleaved caspase-3, BGC-823 cells treated with H2O2 served as the positive control (shown in the right panel). (D) Apoptosis in xenografts was detected by TUNEL labeling (red) and the sections were counterstained with DAPI (blue). Sections from female rodent mammary gland obtained 3∼5 after weaning of rat pups (Millipore) served as the positive control. Original magnification: × 200. Scale bars, 50 µm. ‘R’ represents resveratrol, ‘Ci’ represents the control siRNA, and ‘Si’ represents the Sirt1 siRNA. (TIF) [file pone.0070627.s001.tif]

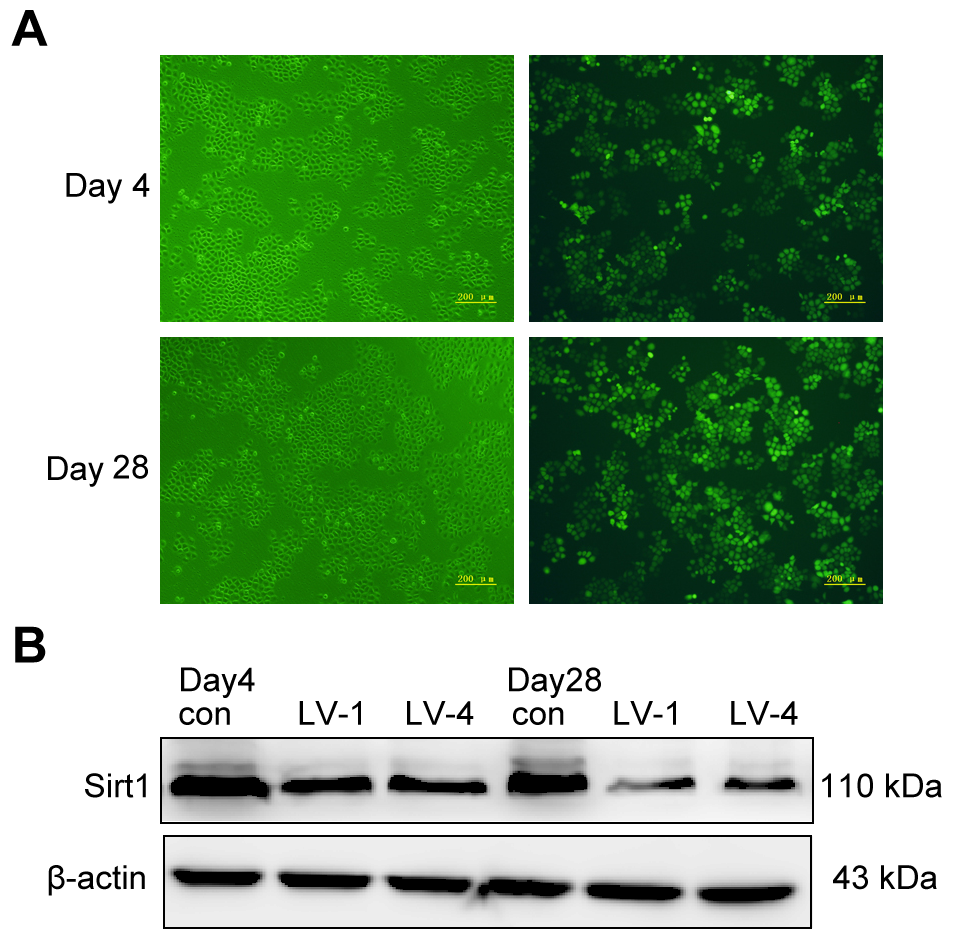

Supplement: Figure S2 — Knockdown of Sirt1 by shRNA-lentivirus. (A) BGC-823 cells were transfected with control or Sirt1-specific shRNA-lentiviruses. The transfection efficiency was evaluated with a fluorescence microscope. At a multiplicity of infection (MOI) of 50, more than 90% of the cells were transfected with shRNA-lentivirus. After four weeks of screening with puromycin, all of the living cells were transduced. Magnification: × 100, bar for 200 µm. (B) The cells were harvested 4 days after infection and 28 days after puromycin screening. The silencing efficiency of Sirt1 was verified by western blot. (TIF) [file pone.0070627.s002.tif]
