# Supplementary material for: Resveratrol Inhibits the Growth of Gastric Cancer by Inducing G1 Phase Arrest and Senescence in a Sirt1-Dependent Manner
Source: PLoS One. 2013 Nov 21;8(11):e70627. doi: 10.1371/journal.pone.0070627 (PMC3836800; doi:10.1371/journal.pone.0070627)
Supplement: Table S1 — Primers for the RT-QPCR experiments performed in this study. (DOC) [file pone.0070627.s003.doc]

**Supplementary Table 1.** Primers for the RT-QPCR experiments performed in this study.

| **Gene** | **Sequence (5’ → 3’)** | **Product (bp)** |
| --- | --- | --- |
| cyclin D1 | AACTACCTGGACCGCTTCCT  CCACTTGAGCTTGTTCACCA | 204 |
| CDK4 | CCAAAGTCAGCCAGCTTGACTGTT  CATGTAGACCAGGACCTAAGGACA | 197 |
| p21 | TCCAGCGACCTTCCTCATCCAC  TCCATAGCCTCTACTGCCA CCATC | 108 |
| β-actin | TTGCCGACAGGATGCAGAA GCCGATCCACACGGAGTACT | 100 |
